# Calcifying epithelial odontogenic tumor of the maxilla

**DOI:** 10.1016/S1808-8694(15)30669-8

**Published:** 2015-10-19

**Authors:** George João Ferreira do Nascimento, Karuza Maria Alves Pereira, Cassiano Francisco Weege Nonaka, Ana Miryam Costa de Medeiros, Hébel Cavalcanti Galvão

**Affiliations:** 1 Master's degree. Doctoral student of the Oral Pathology Graduate Program, UFRN; 2 Master's degree. Doctoral student of the Oral Pathology Graduate Program, UFRN; 3 Master's degree. Doctoral student of the Oral Pathology Graduate Program, UFRN; 4 Doctor in Oral Pathology. Assistant professor of the Oral Diagnosis Discipline, UFRN; 5 Doctor in Oral Pathology. Assistant professor of the Oral Pathology Graduate Program, UFRN. Universidade Federal do Rio Grande do Norte

**Keywords:** maxillary neoplasms, jaw neoplasms, odontogenic tumors

## INTRODUCTION

The calcifying epithelial odontogenic tumor (CEOT) is a rare benign neoplasm; it comprises only 0.6 % to 1.7 % of all odontogenic tumors.^1^, ^2^, ^3^, ^4^ Most cases involve the posterior mandible; there have been few reported maxillary cases.^3^ Although its biological behavior is relatively indolent, maxillary lesions tend to grown rapidly and not be circumscribed.^1^ Treatment of the CEOT consists of surgical removal, ranging from a conservative approach to a more aggressive resection.^4^,^5^ A 14% local recurrence rate has been reported; the prognosis is considered as faborable.^1^, ^2^, ^3^

**Figure 1:**
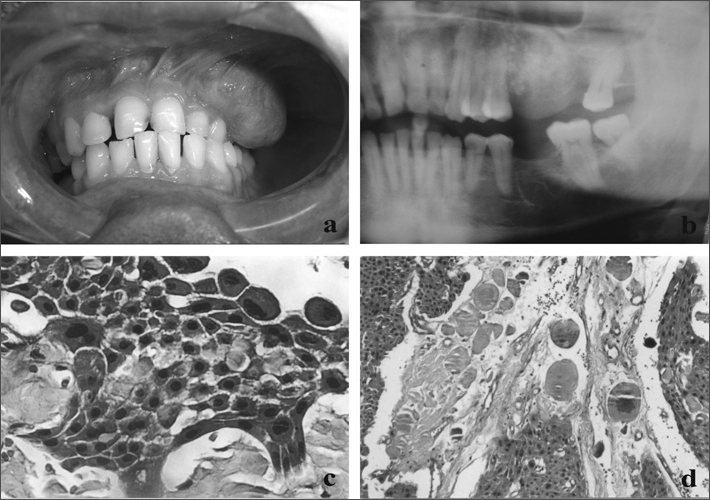
(a) Exophytic nodular lesion in the maxillary vestibular gingiva; (b) panoramic radiograph showing a radiolucent lesion of non-distinct borders and central radiopacities; (c) epithelial polyhedral cell islands with pleomorphism and prominent intercellular bridges (HE/ 400x); (d) concentric lamellar calcifications among epithelial polyhedral cell islands (HE/ 100x).

A male patient aged 35 years visited the Oral Diagnosis Discipline presenting a mass in the left maxilla. Inspection of the mouth revealed a nodule (measuring 8.0 × 5.0 cm) located on the left maxillary vestibular gingival from the lateral incisor to the second molar (Fig. 1a); it had a soft consistency and a lobulated surface.

The patient reported that the lesion had developed within the past 6 months. Radiography revealed a radiolucent area with poorly defined borders and central radiopaque deposits (Fig. 1b). Maxillary vestibular cortical bone expansion and resorption were also evident. These clinical and radiographic findings suggested a calcifying odontogenic cyst or a CEOT.

An incision biopsy was undertaken and the specimen was sent to the Oral Pathology Laboratory. Histopathology showed islands and cords of polyhedral odontogenic epithelial cells; there was cell pleomorphism and prominent intercellular bridges (Fig. 1c), a significant amount of amyloid-like extracellular deposits, and occasional concentric lamellated calcifications (Liesegang's rings) (Fig. 1d), typical of Pindborg's tumor.

Surgery was undertaken, including a marginal portion of apparently healthy bone. One year later there are no signs of recurrence.

## DISCUSSION

CEOT is a rare benign epithelial odontogenic neoplasm; its prevalence ranges from 0.6% to 1.7% of all odontogenic tumors.^1^, ^2^, ^3^, ^4^ This report adds to the small number of Pindborg's tumor cases located in the maxilla, since over 2/3 of CEOT case have been described in the posterior portion of the mandible.^1^, ^2^, ^3^, ^4^, ^5^

Two marked microscopic features were identified in this case: a significant amyloid-like deposit, and sparse mineral deposits with inconspicuous Liesegang's rings. Although there were few mineral deposits in the tumoral stroma, this case was not classified among the rare non-mineralized variants of Pindborg's tumor.^5^,^6^ Furthermore, a relative absence of mineralized areas has been described as typical of peripheral CEOT variants.^1^,^2^

Treatment of CEOT consists of surgical removal, which includes a marginal portion of apparently healthy bone. A minimum 5-year observation period is suggested.^1^,^3^ Maxillary CEOT cases require more aggressive surgery, since these tumors tend to grow more rapidly and are not circumscribed.^1^

Notwithstanding its size and a relatively rapid clinical progression, which suggest a distinct biological behavior of this tumor in the maxilla,^1^ conservative surgery including a non-involved marginal bone area was undertaken in this case. After one year there are no signs of relapse; the patient, however, remains under observation, given the possibility of recurrence within the first five years of surgery.

## FINAL COMMENTS

CEOT is a rare epithelial odontogenic tumor with a marked preference for the mandible; few cases have been reported in the maxilla. In this site, the CEOT tends to grow more rapidly and not be circumscribed, suggesting that more aggressive surgery is required in these specific cases.

One of the typical findings of intraosseous CEOT is the presence of mineral deposits, commonly in the form of Liesegang's rings; extraosseous forms generally do not exhibit these deposits. A paucity of mineral deposits and a maxillary site are the particularities of this case.
